# Comparison of Surgical Site Infection (SSI) Rates in Dogs Undergoing Tibial Plateau Leveling Osteotomy (TPLO) Using Perioperative Versus Peri- and Postoperative Antimicrobial Prophylaxis

**DOI:** 10.3390/vetsci12030258

**Published:** 2025-03-10

**Authors:** Lucrezia Di Filippo, Amanda Bianchi, Andrea Paolini, Umberto Maggiolini, Gert W. Niebauer, Francesco Collivignarelli, Roberto Tamburro

**Affiliations:** 1 Department of Veterinary Medicine, University of Teramo, SP18, 8, Piano d’Accio, 64100 Teramo, Italy; abianchi@unite.it (A.B.); apaolini@unite.it (A.P.); gerhard.niebauer@unina.it (G.W.N.); fcollivignarelli@unite.it (F.C.); rtamburro@unite.it (R.T.); 2 Department of Veterinary Medicine and Animal Productions, University of Naples, Via Federico Delpino 1, 80137 Naples, Italy

**Keywords:** TPLO, antimicrobial prophylaxis, antibiotic resistance, surgical site infection, dog

## Abstract

(1) Background: Postoperative antibiotic prophylaxis in dogs undergoing tibial plateau leveling osteotomy (TPLO) is controversial. We evaluated the effect of perioperative antibiotic administration on the incidence of surgical site infection (SSI) and bone healing in dogs that underwent TPLO procedures and compared this protocol with cases that received prolonged postoperative antibiotic treatment. (2) Methods: Two different antibiotic treatment protocols were used. In group A, cefazolin (22 mg/kg IV) was administered preoperatively 30–60 min before skin incision and repeated every 90–120 min intraoperatively. Antimicrobial prophylaxis was extended in the postoperative period with oral cefazolin (22 mg/kg BID) for 10 days after surgery. In group B, dogs only received perioperative treatment, at the same dose used in group A. Dogs were considered to have SSI in cases of incisional drainage, wound dehiscence, positive bacterial culture, or evidence of radiographic signs of infection. (3) Results: In group A, SSI was observed in 3/61 TPLOs (4.9%). In group B, SSI was present in 3/51 TPLOs (5.9%). There was no difference between the two groups. (4) Conclusions: Our results indicate that perioperative antibiotic prophylaxis in TPLO procedures is sufficient to maintain overall low rates of SSI. The incidence of SSI was similarly low compared to the rate observed in dogs that also received postoperative antibiotic treatment.

## Simple Summary

Tibial plateau leveling osteotomy (TPLO) is a common surgical procedure to treat stifle joints affected by cranial cruciate ligament disease in dogs. To avoid surgical site infection, peri- and postoperative antimicrobial administration is often routinely used. The effectiveness of such prophylactic protocol remains controversial. Tibial plateau leveling osteotomy is a clean orthopedic procedure usually performed in dogs free from other conditions. For these reasons, we hypothesized that perioperative antibiotic treatment alone may be sufficient to maintain surgical site infections within acceptable limits. Dogs undergoing TPLO were divided into two groups with respect to the antibiotic protocol used: in the first group, peri- and postoperative antimicrobial management was used, while in the second group, only perioperative antibiosis was administered. Results showed no difference in surgical site infection rates between the two groups, suggesting that extending antibiotic use in the postoperative period is unnecessary. Eliminating postoperative antibiotic medication in a clean orthopedic procedure such as TPLO helps to reduce antibiotic resistance and is in line with the generally accepted standards of antimicrobial stewardship.

## 1. Introduction

Tibial plateau leveling osteotomy (TPLO) is a widely used surgical procedure to stabilize the stifle joint in dogs affected by cranial cruciate ligament disease [1,2,3,4]. The aim of the surgery is to provide a 90° angle between the patellar tendon and the tibial plateau in order to neutralize the tibial thrust. Thus, a proximal circular radial osteotomy is performed to rotate the osteotomized proximal tibia counterclockwise, thereby reducing the tibial plateau angle. The osteotomy is then transfixed with a specific pre-angled locking plate [1,2,3,4].

Complications associated with TPLO include infection, seroma, intraoperative or delayed cranial tibial artery hemorrhage, soft and bone tissue complications, and implant failure, resulting in an overall complication rate ranging from 15% to 37% [5,6,7,8].

TPLO surgery has been associated with an average risk of surgical site infection (SSI), reported to be between 0.8% and 14.3% [7]. Reasons for the relatively higher rate in some reports are unclear and are likely multifactorial. Potential factors contributing to SSI include thermal damage by the oscillating saw blade, sparse soft-tissue coverage of the proximal aspect of the tibia, extensive soft tissue and periosteal dissection, prolonged surgery and anesthesia times, and increasing prevalence of opportunistic pathogens (particularly staphylococci) that are often resistant to the commonly used antimicrobials for perioperative prophylaxis [9,10]. Infection may lead to pain, lameness, delayed healing, and potential surgical failure. Thus, antibiotic perioperative and postoperative prophylaxis is a core strategy for the prevention of surgical site infections (SSI) [11,12]. Standard protocols include IV administration of cefazolin 30–60 min before the skin incision, repeated every 90–120 min, intraoperatively. Postoperatively, cefazolin and/or amoxicillin/clavulanic acid may be administrated for up to 7–10 days [11].

Tibial plateau leveling osteotomy is a clean orthopedic procedure that uses metallic implants. Therefore, standard protocols to avoid SSI, which are generally limited to perioperative antibiosis, should be applied. Avoiding postoperative prophylactic antibiosis in uncomplicated TPLO procedures is a generally accepted concept nowadays. Although clinical experience seems to support this concept, published evidence regarding TPLO procedures is lacking.

The aim of this study was to retrospectively analyze the SSI rate in a cohort of dogs that underwent TPLO surgery at the same institution by the same surgical team. The cohort was divided into two groups in two different time periods with two different prophylactic antibiotic protocols: in the first period (2017–2019), the treatment protocol consisted of perioperative and postoperative antibiotic administration, while in the second period (2020–2022), only perioperative prophylaxis was used. We hypothesized that perioperative antibiotic prophylaxis alone may be sufficient to maintain an overall low SSI rate.

## 2. Materials and Methods

The retrospective study included all consecutive cases of TPLO performed at the Veterinary Teaching Hospital of the University of Teramo (SP18, 8, 64100 Piano d’Accio, TE) between May 2017 and June 2022.

The animal study protocol was approved by the Ethics Committee of the University of Teramo (protocol code 24520, 6 October 2021).

All dog owners signed an informed consent prior to surgery. Animals in which the following were recorded were excluded from the study: potentially contaminated skin lesions on the affected legs, metabolic and/or infectious diseases, previous treatments with any antimicrobials and/or corticosteroids, and dogs with compromised cardiac function.

Medical records of dogs that underwent TPLO surgery were reviewed. Timing and dosage of antimicrobial administration, duration of the surgical procedures and anesthesia, evolution and outcome of SSI, microbiological investigation, implant removal, complications and any comorbidities were analyzed.

Dogs received two different antibiotic treatment protocols. From March 2017 to December 2019 (group A), cefazolin (22 mg/kg IV) was administered preoperatively 30–60 min before skin incision and repeated every 90–120 min intraoperatively, depending on the length of surgery. In addition, antimicrobial prophylaxis was extended over a 10-day-long postoperative period with cefazolin (22 mg/kg BID per OS) [13].

From January 2020 to April 2022 (group B), dogs received cefazolin (22 mg/kg IV) only pre- and perioperatively following the identical protocol used in group A, but without continued postoperative antimicrobial prophylaxis.

For surgery, dogs were premedicated with methadone (0.2 mg/kg) and dexmedetomidine (5 mcg/kg IM), then induced for endotracheal intubation with propofol (4 mg/kg) and maintained on isoflurane in oxygen [14]. Hemodynamically stable patients received continuous infusion of dexmedetomidine (0.5 mcg/kg/h) [14]. At the induction time, a dose of cefazolin (22 mg/kg IV) was administrated, and dogs were routinely prepared for aseptic surgery. A typical TPLO approach was performed on the medial aspect of the affected limb. A medial stifle arthrotomy was performed to assess the condition of the stifle joint, and to debride the residual portion of the cranial cruciate ligament rupture (CCLr) and to treat potential meniscal damages. Then, a radial osteotomy was performed and fixed according to Slocum’s TPLO technique [1]. During the procedure, any hemorrhage was carefully controlled, with special attention to hemostasis, especially during the use of the oscillating saw, where abundant irrigation was applied. At the end of the procedure, a final lavage with sterile saline solution was carried out, followed by a culture swab of the surgical field around the implants. Additionally, care was taken in closing, avoiding any dead spaces. The fascial plane and subcutis were affixed with a continuous pattern using absorbable monofilament material (USP 2-0 or 3-0), and the skin was closed with interrupted sutures or a continuous pattern using non-absorbable material (USP 2-0 or 3-0). The surgical wound was cleaned with 0.9% chlorhexidine solution and covered with a modified Robert Jones bandage. All TPLO procedures were performed by the same surgeon (RT). Postoperative radiographic exams were performed verifying appropriate reduction and positioning of the implants. A modified Robert Jones bandage was applied for 48 h after surgery.

All patients were discharged the day after surgery with an Elizabethan collar until skin suture removal. Owners were instructed to keep dogs on rest for 4 weeks, allowing leach walking for 5–10 min three times a day. After bandage removal, wound care, including topical disinfection three times a day, was carried out by the owners for twelve days after surgery.

Dogs received meloxicam at 0.1 mg/kg SID for 4 weeks, and postoperative re-examinations were performed at 14 days, 4 weeks, and 8 to 12 weeks. Surgical wounds were checked on the second and fourteenth day after surgery. A radiographic follow up was performed up to 12 weeks post-surgery using mediolateral and craniocaudal projections.

Dogs were considered affected by surgical site infection (SSI) if they met any of the following criteria: incisional drainage (the presence of abnormal fluid or pus from the surgical site, which required clinical intervention); wound dehiscence (partial or complete separation of the wound margins that required additional medical or surgical management); positive bacterial culture (a positive culture obtained from the surgical site, identifying an infection-causing microorganism); radiographic signs of SSI (evidence of infection or delayed bone healing on radiographic images, such as the presence of sequestrum or osteolysis).

Dogs were considered free of SSI if they exhibited normal wound healing and no radiographic signs that indicate delayed osteotomy closure.

Cases of confirmed SSI in both groups (A and B) were compared using Pearson’s chi-squared test. The significance level was set at *p* < 0.05.

## 3. Results

A total of 102 dogs and 112 TPLO procedures met the inclusion criteria for the study. Group A comprised 59 dogs (61 TPLO procedures), while group B consisted of 41 dogs (51 TPLO procedures).

Group A included 36 females and 23 males. The median body weight was 23 kg (range 4–60 kg), and the median age was 6 years (range 1–10). Group B included 28 females and 13 males. The median body weight was 19 kg (range 3–55 kg), and the median age was 5 years (range 2–11).

Surgical site infections (SSI) were observed in 3 TPLOs out of 61 in group A, resulting in an incidence of 4.9%.

**Figure 1:**
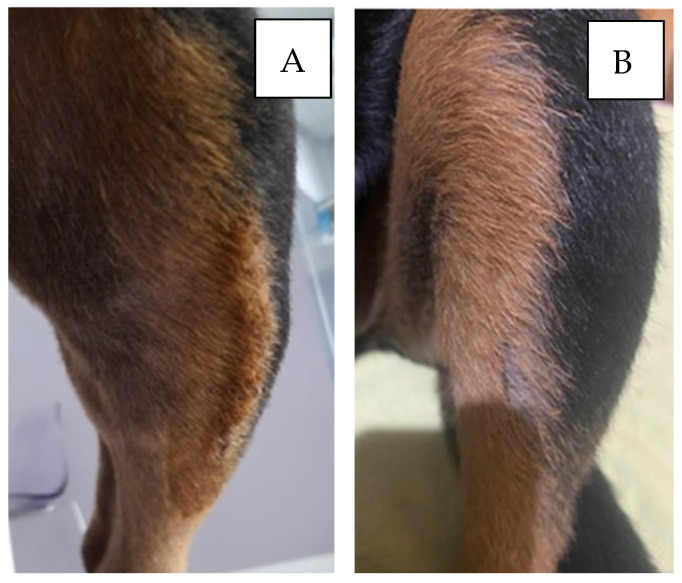
Dog: Rottweiler, f, 6 years old, left TPLO. Wound assessment performed postoperatively 12 (**A**) and 30 (**B**) days after surgery. No evidence of wound infection was observed.

In group B, SSI occurred in 3 TPLOs out of 51, with an incidence of 5.9%. All intraoperative culture swabs from both groups were negative (Figure 1).

The incidence of SSI was similar in both groups, with no statistically significant difference (*p* > 0.05).

**Figure 2:**
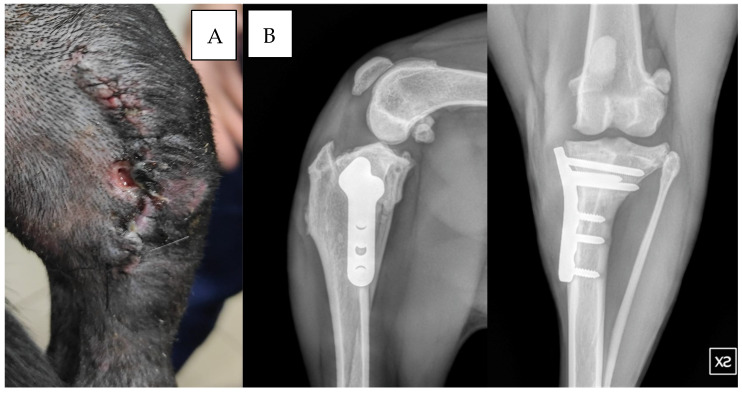
Dog: Pitt bull, f, 5 years old, left TPLO. Wound dehiscence was observed 10 days after surgery. Staphylococcus hominis ssp. was isolated from the culture swab (**A**). The two radiographic projections show the control 90 days after surgery, note the absence of any signs of SSI (**B**).

In group B, one dog exhibited wound dehiscence 10 days post-surgery (Figure 2). Staphylococcus hominis was cultured from the wound, and sensitivity testing showed positive results for enrofloxacin but resistance to cefazolin, and amoxicillin/clavulanic acid was present.

**Figure 3:**
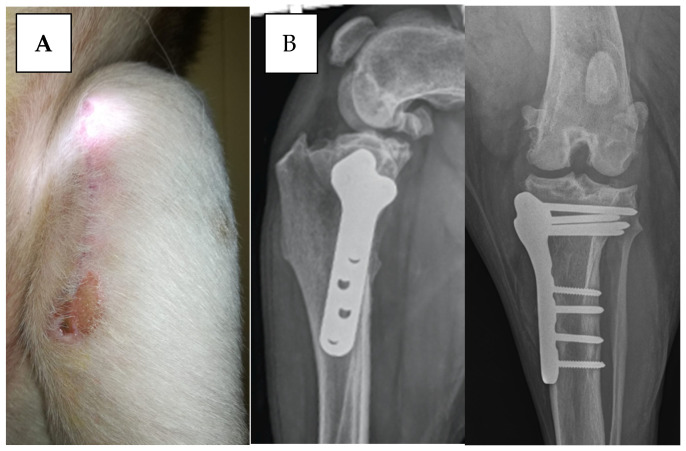
Dog: mixed breed, m, 5 years old, 55 kg, left TPLO. Wound dehiscence was observed 10 days after surgery. Serratia marcescens was isolated from the culture swab (**A**). The two radiographic projections show the control 90 days after surgery, demonstrating that there are no signs of SSI (**B**).

In another dog from group B, wound discharge was noted 15 days post-surgery. In a third case from group B, the culture revealed Serratia marcescens (Figure 3A). The antibiogram showed sensitivity to enrofloxacin and marbofloxacin, while resistance to cefazolin and amoxicillin/clavulanic acid was observed.

Dogs with wound dehiscence underwent surgical wound cleansing and received 20 days of oral antibiotics (enrofloxacin 5 mg/kg BID). Radiographs revealed ossification 90 to 100 days post-surgery (Figure 3B).

The overall findings of the study, including SSI incidence and complications, are summarized in Table 1.

In group A, one dog had a wound dehiscence 10 days after surgery, and in two cases, a fistula developed two years after surgery. Implant removal was performed in the latter, which resolved the complication.

The time from skin incision to bacteriologic sampling at the end of procedures ranged from 70 to 118 min. In 23 cases, surgical time exceeded 90 min, and therefore two doses of cefazolin (22 mg/kg) were administered intraoperatively. In the remaining cases, a single dose was given.

## 4. Discussion

The prophylactic use of antimicrobics during orthopedic surgical procedures aims at reducing or avoiding SSI. Surgical site infections are an intrinsic risk of every surgery, leading to increased morbidity and mortality and are potentially costly complications.

The efficacy of postoperative antibiotic treatment in dogs undergoing TPLO surgery is controversial. In a study involving 659 TPLO procedures on 541 dogs, in 71 of 659 (11%) cases, SSI infections were found; in 20 of those 71, (28%) methicillin-resistant Staphylococcus pseudintermedius was present. Based on these findings, it was concluded that early administration of perioperative and postoperative antimicrobial administration may improve protection against SSI after TPLO [15]. In another report, the outcome of 93 consecutive clean orthopedic procedures was evaluated; 72 out of 93 were TPLOs. Dogs were divided into two groups: those receiving postoperative antibiosis, and those receiving perioperative antibiosis alone. Post-TPLO infection was detected in two dogs that received perioperative and postoperative oral antimicrobials for 10 days, and in five dogs receiving only perioperative antimicrobials [11]. It was concluded that although postoperative antimicrobial treatment is not recommended for humans, the pre- and postoperative environment and surgical facilities in human hospitals may not be entirely comparable with those in animal hospitals. Patient interference with wounds is one factor that is likely to be more important in animals. Therefore, well-established protocols in human medicine should be cautiously extrapolated in animals [11]. In 2020, a clinical study determined whether extending prophylactic antimicrobial administration into the postoperative period would decrease the surgical site infection (SSI) rate in clean canine orthopedic surgery associated with metal implants. Regarding TPLOs, during a 6-week postoperative observation period, infections were observed in a total of 8/117 in the perioperative antibiosis group compared with 4/123 in the perioperative and postoperative antibiotic treatment group. On long-term follow-up, infections were reported in six out of sixty-four TPLOs and in five out of fifty-two TPLOs in the perioperative group versus the postoperative group, respectively, which demonstrated that continued postoperative antibiosis had no effect on SSI [16].

On the other hand, a recent study evaluated the outcomes for dogs that did not receive antimicrobial medications and dogs that did receive oral antimicrobial medications for 14 days after TPLO, concluding that postoperative administration of antimicrobial medications was not protective against SSI [17]. Similarly, a more recent investigation found no significant difference in the incidence of SSI between two groups (17% vs. 11%, with *p* = 0.34), indicating that cefpodoxime did not substantially reduce the SSI risk in dogs undergoing TPLO [13]. This supports the ongoing uncertainty regarding the efficacy of postoperative antibiotics in preventing SSI in these cases. In a systematic review of studies in which the antibiotic treatment and infection rates were evaluated and reported, the overall conclusion was that the efficacy of postoperative antibiotic treatment in dogs affected by TPLO remains inconclusive [18].

Additionally, a study reviewing the outcomes of implant removal in dogs with SSI after TPLO found that treatment with antibiotics alone was unsuccessful in 88.9% (64/72) of the dogs. Implant removal, with or without postoperative antibiotic therapy, led to the resolution of clinical signs in 94.9% (74/78) of the cases, demonstrating that antibiotic treatment without implant removal is largely ineffective [9]. The presence of a metallic implant, after completed bone healing, represents a potential source of infection, even in the long term. However, implant removal requires another surgical procedure, which may have inherent risks. For this reason, we removed implants only when there was evidence of infection, such as the presence of fistulae.

Another study evaluated the rate of implant-associated infections in dogs undergoing TPLO, either with a standard protocol or a revised antiseptic protocol. Implant-associated infections were reduced by optimizing antibiotic administration, using antimicrobial drapes, using improved surgical gloves, and by adhering to postoperative care protocols, such as topical mupirocin application and Elizabethan collar use. These changes led to a significant reduction in infection rates from 7.4% to 0.94% [13]. In our study, we did not use such additional measures (antimicrobial drapes or mupirocin); however, we did not observe a significant difference in infection rates.

A recent study on intraoperative antiseptic lavage, however, found that using an antiseptic lavage solution (instead of saline) during TPLO surgery did not significantly reduce the incidence of SSIs. In fact, the antiseptic lavage group showed a higher infection rate (14.8%) compared to the saline group (8.9%), with no substantial difference in the likelihood of developing an SSI [19]. In our study, we performed extensive saline lavage (without antiseptics) throughout the procedure, ensuring copious tissue irrigation, particularly to prevent infection related to thermal necrosis as a sequel to using powered sawing and drilling equipment.

Centers for Disease Control and Prevention guidelines suggest that antibiotic administration should be initiated before skin incision in order to obtain sufficient antibiotic tissue levels during surgery. In addition, inappropriate antibiotic treatment may have no effects, leading to systemic toxicity and multidrug resistance.

Following this principle, we decided to change our approach to antibiotic stewardship. Preoperative and postoperative antibiotics were routinely used in the first period. Since 2020, considering that TPLO is a clean surgical procedure, we have assumed that perioperative antibiotic treatment may be sufficient to yield an acceptably low SSI rate. For this reason, we adapted our protocol, eliminating postoperative prophylaxis and applying only perioperative antibiotic treatment after TPLO.

According to our results, no statistical differences have been observed between the outcomes in the two treatment groups. In the dogs receiving perioperative antibiotic administration only (group B), all bacterial culture swabs, taken at the end of the procedure, were negative. Nevertheless, two of those dogs developed a superficial SSI infection ten and fifteen days after surgery, respectively. This suggests that culture swabs should be considered as a screening test only. In both cases, the cultured bacteria were sensitive to enrofloxacin and resistant to cefalexin, the standard antibiotic treatments for orthopedic surgery. Treatment with enrofloxacin was resolutive in both cases.

The third case of SSI (group B) was observed two years after surgery and a fistulous tract had developed, suggesting a deep surgical site infection. In this case, implant removal was performed. As the infection occurred two years postoperatively, we hypothesized that it was a consequence of hematogenous bacterial dissemination that colonized the implant, producing a biofilm. Our results are congruent with two previous studies which similarly found that postoperative antibiotics do not alter surgical site infection rates after TPLO surgery [13,16,17,20].

## 5. Conclusions

According to our results, perioperative antibiotic prophylaxis alone is sufficient to maintain a low rate of surgical site infections (SSI) following TPLO surgery. The SSI rate in dogs receiving only perioperative antibiotics (cefalexin) was comparable to that of dogs receiving additional postoperative cefalexin therapy, suggesting that extended postoperative antibiotic treatment may not be necessary to prevent SSI in these cases.

The use of postoperative antibiotics in clean orthopedic surgeries still remains a topic of debate. Further studies are certainly required to address this issue. However, our findings provide data clearly demonstrating that in this type of surgery, the risk/benefit ratio is strongly in favor of not using postoperative antibiotics. Instead, relying on perioperative prophylaxis only and on strict adherence to aseptic surgical techniques seems sufficient for controlling surgical site infections, and last but not least, constitutes a valuable contribution to antibiotic stewardship.

## Figures and Tables

**Table 1:** Summary of SSI incidence and complications in group A and group B.

Group	Number of TPLOs	Number of Dogs	SSI Incidence	Complications
**Group A** **(Cefazolin pre- and postoperative protocol)**	61	59	4.9% (3 TPLOs with SSI)	1 case developed wound dehiscence. 2 cases developed a fistula.
**Group B** **(Cefazolin preoperative protocol only)**	51	41	5.9% (3 TPLOs with SSI)	2 cases developed wound dehiscence. 1 dog developed a fistula two years after surgery.
**Total**	112	100	5.4% (6 TPLOs with SSI)	3 cases of wound dehiscence, 3 cases of fistula.

## Data Availability

The original contributions presented in this study are included in the article. Further inquiries can be directed to the corresponding authors.

## Abbreviations

The following abbreviations are used in this manuscript:

TPLO	Tibial plateau levelling osteotomy
SSI	Surgical site infection

## References

[1] Slocum B., Slocum T.D. (1993). Tibial Plateau Leveling Osteotomy for Repair of Cranial Cruciate Ligament Rupture in the Canine. Vet. Clin. N. Am. Small Anim. Pract..

[2] Vezzoni L., Bazzo S., Boiocchi S., Vezzoni A. (2020). Use of a Modified Tibial Plateau Levelling Osteotomy with Double Cut and Medial Crescentic Closing Wedge Osteotomy to Treat Dogs with Cranial Cruciate Ligament Rupture and Tibial Valgus Deformity. Vet. Comp. Orthop. Traumatol..

[3] Amimoto H., Koreeda T., Ochi Y., Kimura R., Akiyoshi H., Nishida H., Miyabayashi T., Beale B.S., Hayashi K., Wada N. (2020). Force Plate Gait Analysis and Clinical Results after Tibial Plateau Levelling Osteotomy for Cranial Cruciate Ligament Rupture in Small Breed Dogs. Vet. Comp. Orthop. Traumatol..

[4] Cosenza G., Reif U., Martini F.M. (2015). Tibial Plateau Levelling Osteotomy in 69 Small Breed Dogs Using Conically Coupled 1.9/2.5 mm Locking Plates. A Clinical and Radiographic Retrospective Assessment. Vet. Comp. Orthop. Traumatol..

[5] Gallagher A.D., Mertens W.D. (2012). Implant Removal Rate from Infection after Tibial Plateau Leveling Osteotomy in Dogs. Vet. Surg..

[6] Ferrari F., Tamburro R., Longo M., Brioschi F.A., Auletta L., Stefanello D. (2024). Effect of cranial tibial artery laceration on radiographic bone healing and perioperative complications in dogs undergoing tibial plateau leveling osteotomy. Res. Vet. Sci..

[7] Bergh M.S., Peirone B. (2012). Complications of Tibial Plateau Levelling Osteotomy in Dogs. Vet. Comp. Orthop. Traumatol..

[8] Grand J.-G. (2024). Delayed life-threatening hemorrhage caused by cranial tibial artery Pseudoaneurysm in two dogs. J. Am. Anim. Hosp. Assoc..

[9] Savicky R., Beale B., Murtaugh R., Swiderski-Hazlett J., Unis M. (2013). Outcome Following Removal of TPLO Implants with Surgical Site Infection. Vet. Comp. Orthop. Traumatol..

[10] Nazarali A., Singh A., Weese J.S. (2014). Perioperative Administration of Antimicrobials During Tibial Plateau Leveling Osteotomy: Perioperative Administration of Antimicrobials During TPLO. Vet. Surg..

[11] Pratesi A., Moores A.P., Downes C., Grierson J., Maddox T.W. (2015). Efficacy of Postoperative Antimicrobial Use for Clean Orthopedic Implant Surgery in Dogs: A Prospective Randomized Study in 100 Consecutive Cases. Vet. Surg..

[12] Ailaney N., Zielinski E., Doll M., Bearman G.M., Kates S.L., Golladay G.J. (2021). Variation in Practice for Preoperative Antibiotic Prophylaxis: A Survey from an Academic Tertiary Referral Center in the United States. Patient Saf. Surg..

[13] Spencer D.D., Daye R.M. (2018). A prospective, randomized, double-blinded, placebo-controlled clinical study on Postoperative Antibiotherapy in 150 arthroscopy-assisted tibial plateau leveling osteotomies in dogs. Vet. Surg..

[14] Duke-Novakovski T., Vries M., de Seymour C. (2020). BSAVA Manual of Canine and Feline Anaesthesia and Analgesia.

[15] Hagen C.R.M., Singh A., Weese J.S., Marshall Q., Linden A.Z., Gibson T.W.G. (2020). Contributing Factors to Surgical Site Infection after Tibial Plateau Leveling Osteotomy: A Follow-up Retrospective Study. Vet. Surg..

[16] Aiken M.J., Hughes T.K., Abercromby R.H., Holmes M.A., Anderson A.A. (2015). Prospective, Randomized Comparison of the Effect of Two Antimicrobial Regimes on Surgical Site Infection Rate in Dogs Undergoing Orthopedic Implant Surgery. Vet. Surg..

[17] Clark A.C., Greco J.J., Bergman P.J. (2020). Influence of Administration of Antimicrobial Medications after Tibial Plateau Leveling Osteotomy on Surgical Site Infections: A Retrospective Study of 308 Dogs. Vet. Surg..

[18] Budsberg S.C., Torres B.T., Sandberg G.S. (2021). Efficacy of Postoperative Antibiotic Use after Tibial Plateau Leveling Osteotomy in Dogs: A Systematic Review. Vet. Surg..

[19] Sanders B.D., McDonald-Lynch M., Kruse M.A. (2023). Influence of antiseptic lavage during tibial plateau leveling osteotomies on surgical site infection in 1422 dogs. Vet. Surg..

[20] Stine S.L., Odum S.M., Mertens W.D. (2018). Protocol changes to reduce implant-associated infection rate after tibial plateau leveling osteotomy: 703 dogs, 811 TPLO (2006–2014). Vet. Surg..

